# Helium Atmospheric Pressure Plasma Jet Effects on Two Cultivars of *Triticum aestivum* L.

**DOI:** 10.3390/foods12010208

**Published:** 2023-01-03

**Authors:** Ion Burducea, Cristina Burducea, Paul-Emil Mereuta, Stefan-Robert Sirbu, Decebal-Alexandru Iancu, Melania-Beatrice Istrati, Mihai Straticiuc, Constantin Lungoci, Vasile Stoleru, Gabriel-Ciprian Teliban, Teodor Robu, Marian Burducea, Andrei Vasile Nastuta

**Affiliations:** 1 “Horia Hulubei” National Institute for Research & Development in Physics and Nuclear Engineering, 077125 Magurele, Romania; 2 Department of Plant Science, Iasi University of Life Sciences, 3 Sadoveanu Alley, 700490 Iasi, Romania; 3 Department of Horticulture, Iasi University of Life Sciences, 3 Sadoveanu Alley, 700490 Iasi, Romania; 4 Research and Development Station for Aquaculture and Aquatic Ecology, “Alexandru Ioan Cuza” University, Carol I, 20A, 700505 Iasi, Romania; 5 Physics and Biophysics Education Research Laboratory (P&B-EduResLab), Biomedical Science Department, Faculty of Medical Bioengineering, ‘Grigore T. Popa’ University of Medicine and Pharmacy Iasi, Str. M. Kogalniceanu No. 9-13, 700454 Iasi, Romania

**Keywords:** plasma agriculture, atmospheric-pressure plasma, seed morphology, germination, biochemical analyses, chlorophyll, polyphenols, antioxidant activity, nutrients

## Abstract

The use of cold plasma in the treatment of seeds before sowing presents a promising technique for sustainable agriculture. The objective of this study is to evaluate the effect of cold plasma treatment on the morphology of wheat seeds (*Triticum aestivum* L. ‘Dacic’ and ‘Otilia’), their germination, biochemical composition, and the nutritional quality of wheat grass. Wheat seeds were morphologically and elementally characterized by atomic force microscopy (AFM), scanning electron microscopy coupled with energy dispersive X-ray spectroscopy (SEM-EDX), X-ray computer tomography (CT), and particle-induced X-ray emission (PIXE). Helium was used as a working gas for plasma generation and the analysis of the species produced showed the presence of NO_γ_, OH, N_2_ and N_2_^+^ and O. Evaluation of germination and plant growth for 10 days (wheat grass stage) highlighted a specific trend for each cultivar. The biochemical analysis of wheat grass highlighted an increase in the chlorophyll content in the plasma-treated variants, an increase in the flavonoid and polyphenol content in ‘Dacic’-treated variant, while the soluble protein content, antioxidant activity, and color were not affected. The analysis of the nutritional quality of wheat grass by the FT-NIR analytical technique highlighted an increase in the ash content in the plasma-treated wheat cultivars, while the humidity, proteins, neutral detergent fiber (NDF), acid detergent fiber (ADF), and energy values were not affected.

## 1. Introduction

The United Nations’ sustainable development goal 2, “zero hunger” mission to eliminate hunger and ensure food security while increasing the quality of food for the population and promoting sustainable agriculture, is strongly affected by recent global events such as the COVID-19 pandemic, the war in Ukraine, and climate change [1,2,3]. At the same time, the world’s population has recently reached eight billion inhabitants and continues to grow, so ensuring food security by increasing crop production is of utmost importance. In this context, research on improving agricultural crops through unconventional, innovative technologies is being explored. Plasma agriculture is a field of research that is in full development due to the vast potential of improving agricultural crops through a non-polluting method compared to conventional agriculture [4]. The most well-known usage of plasma in agriculture is as a seed-germination inducer or even as a green fertilizer. This field is based on the use of cold plasma for the treatment of seeds, seedlings, and soil in order to obtain larger and healthier yields [5]. In addition to the mentioned effects, plasma treatments can accelerate germination, increase the length of plants and their roots, as well as the number of root branches, determine a more efficient use of water, and increase the resistance of plants to diseases [6]. Many studies concerning plasma treatments of various types of seeds, plants, and liquid media can be found in the literature, each showing the benefits of using plasma technology in various ways on the biological material [7,8,9]. Cold plasmas are electric discharges in gases (usually He, Ar, N_2_, O_2_, or their mixtures) that usually have the gas temperature below 100 °C. The development of atmospheric-pressure plasmas, which usually are related to discharges in open environment or in close containers but at elevated pressure to atmospheric pressure, with no vacuum system required, has opened up new possibilities including medicine, food industry, materials, and even the automotive industry, with results being similar to low-pressure plasmas in terms of efficiency, but less expensive [10,11]. In agriculture, the main advantage of using atmospheric-pressure plasma is the fact that the seeds are no longer exposed to a vacuum, so no additional costs are needed [4]. However, the effects on biological systems are dependent on the plasma composition, time of exposure to the treatment, the size, and structure of the targeted biologic material [12]. The composition of the plasma is complex and includes ultraviolet radiation, ions, and electrons; for this reason it is difficult to identify precisely which component determines the greatest effect [12]. Moreover, due to the fact that a plasma discharge source at atmospheric pressure usually means that, besides the working gas, we encounter also plasma-excited species, more likely due to open air or due to controlled addition, it is of interest to identify and monitor, and further tailor plasma parameters in such way to produce a specific amount of plasma-reactive nitrogen and oxygen species, also known as RONS [13]. Plasma RONS play an important role in the plant’s reaction mechanism activating physiological processes that promote seed germination and vegetative growth [6]. Furthermore, plasma-reactive species are involved in the inactivation of microorganisms that are frequently observed in agricultural applications, through oxidation of proteins, lipids, and nucleic acids [14]. If the involvement of such plasma-excited species in interaction with polymeric materials, metals, and even living tissue is known in the literature, their effects on plants are still under study, all of which support ongoing studies [15]. In general, the mechanisms of action that determines the biological effects are influenced by the physical, chemical, and biochemical changes undergone by the seeds as a result of exposure to the plasma treatment [6]. Positive effects of plasma treatments on plants can be manifested in both primary metabolism (growth and development) and/or on secondary metabolism (synthesis of bioactive compounds with a protective role). For example, Ling et al. (2014) [16] showed that treatment with cold He plasma-stimulated germination and seedling growth (shoot length, shoot dry weight, root length, and root dry weight) in *Glycine max*, while Mildaziene et al. (2017) [17] showed that treatment with cold plasma stimulated the synthesis of bioactive compounds (chicoric acid and vitamin C) in *Echinacea purpurea*.

Wheat is one of the most important cereals globally [18]. It is used both for human food and animal feed, and the germinated seeds can be consumed as wheat germs, while wheat grass juice is obtained from the young plants called wheat grass. Due to its global importance, numerous varieties and hybrids with varied morphophysiological characteristics have been created to adapt to various local environmental conditions and to resist to diseases and pests [19,20].

Our approach consists of directly exposing wheat seeds to an atmospheric pressure plasma jet and monitoring and interpreting the resulting effects from the perspective of the morpho-anatomy and physiology of the resulting plants. Using a series of techniques and methods for surface study (AFM, SEM-EDX, CT, and PIXE), as well as various biochemical methods and analytical techniques (UV-VIS and FT-NIR) to follow the aspects of the surface of the wheat seeds and parameters related to the plant growth and nutritional composition of young plants (wheat grass stage) resulting from the germination of the seeds.

## 2. Materials and Methods

The section is divided into two parts: one related to the experimental arrangement and methods used for plasma-source ignition, characterization, and treatment—Section 2.1; and the second one that includes the organic material (wheat seed) exposed to plasma treatment, as well as the physico-chemical methods used for wheat seeds and wheat grass characterization—Section 2.2.

For the present study, seeds of two wheat cultivars (*Triticum aestivum* L.),’Dacic’ and ’Otilia’, were procured from Ezareni research station of Iasi University of Life Sciences. ‘Dacic’ is produced at SCDA Lovrin and ‘Otilia’ at INCDA Fundulea, Romania. Four experimental variants were made (V1 ‘Dacic’ control, V2 ‘Dacic’ treated, V3 ‘Otilia’ control, V4 ‘Otilia’ treated), each containing 100 seeds.

### 2.1. Plasma Source and Electro-Optical Diagnosis

**Figure 1:**
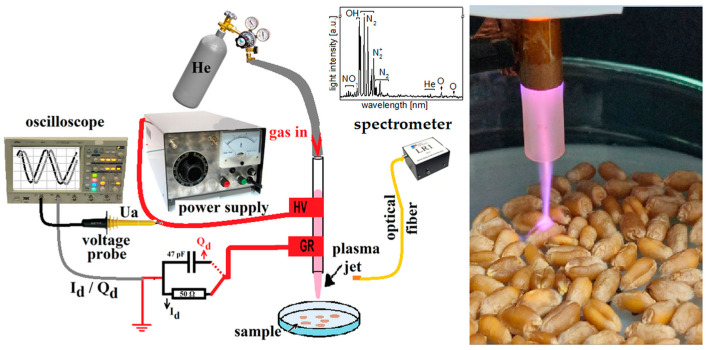
Experimental set-up (**left**) and a photograph (**right**) of the plasma source in interaction with the wheat seed samples and the electro-optical diagnosis arrangement.

An atmospheric pressure plasma jet was used to treat wheat seeds, as shown in Figure 1. Helium was used as the feed gas, flowing through the plasma jet quartz tube at a flow rate of 1.5 standard liters per minute. The high voltage and ground electrodes were wrapped on the exterior of the tube at a gap of 10 mm, the ground one being at 5 mm away from the tube edge. A 10 mm gap between the discharge tube and the Petri dish containing the seeds was maintained during the experiments. The Petri dish (9 cm in diameter) containing 100 wheat grass seeds acts as the grounded electrode. The power supply generated a bipolar AC output with a peak voltage of 0–20 kV at a frequency of 48 kHz. The discharge power was calculated using a Lissajous figure formed with the charges across the capacitor and the applied voltage across the discharge. In this study, all plasma treatments of wheat grass seeds were performed using an atmospheric plasma jet at an applied voltage of up to 14 kV. The Petri dishes were then exposed to He plasma, based on dielectric barrier discharge principles (DBD), for 5 min and continuously rotated so that the plasma jet covered all the seeds in the Petri dish. Meanwhile, the same number of seeds in the control group were placed in similar Petri dishes for germination.

The plasma source used in these studies consisted of a quartz tube (inner and outer diameters of 4 and 6.1 mm) and two 10 mm wide copper tape electrodes, separated by a 10 mm gap. One electrode was connected to the high voltage output of the power supply, the high voltage electrode (HV), and the other one was connected to ground, the grounded electrode (Gr), both wrapped on the exterior of the quartz tube, similar to that reported by Huzum and Nastuta (2021) [11]. The discharge was driven by a 1–40 kV, 20–70 kHz frequency, 10–300 W adjustable power supply (PVM500, Information Unlimited, Mont Vernon, NH, USA). The applied sinusoidal voltage U_a_ (up to 18 kVpp, @ 48 kHz) and the total current of the discharge I_d_, were monitored using voltage and current probes (Testec HVP-15HF, Lecroy PP006A, 50 Ω charge resistor, a 47 pF charge capacitor) and a 350 MHz digital oscilloscope (Lecroy Wavejet 334, four channels, 2 GS/s). The working gas was helium (He 5.0, Siad, Romania), fed through the discharge tube at a fixed flow rate of 1.5 slm, using a needle valve rotameter (Platon, NGVS312 series, France). The spectral emission of the discharge in the UV-to-NIR range (200–900 nm) was analyzed by using a broad range spectrometer (LR1 monochromator, 50 µm entrance slit, 600 gr/mm diffraction grating, ASEQ Instruments, Vancouver, BC, Canada), via a 0.4 mm diameter and 1 m long optical fiber (200–1400 nm, Thorlabs, NJ, USA) and placed at 5 mm from the plasma.

### 2.2. Seed Characterization, Germination, and Plant Growth Parameters

After applying the plasma treatment, 90 seeds from each variant were transferred into nine Petri dishes per variant (10 seeds per dish) for germination and growth evaluation and 10 seeds were used for micromorphological characterization.

#### 2.2.1. Wheat Grass Characterization: Germination and Plant Growth

The samples were incubated at a constant temperature of 22 °C in the dark for 3 days, after that they were illuminated with a commercial white LED band (7.2 W m^−1^) light source. During germination and growth, 5 mL of water were added to each Petri dish every day to maintain moisture.

The germination percentage (GP) was recorded after 3 days (GP = seeds germinated/total seeds × 100).

The morphological measurements of wheat plants were performed 10 days after germination. The total length of wheat grass, including the length of the plant and the length of the root, was measured with a ruler.

Fresh biomass was determined with an analytical balance, and the water content was determined after oven drying of plants at 105 °C until constant weight and expressed in percentage (%).

The leaf area was determined for 45 plants per variant with LI-3100C area meter, LI-COR, (Lincoln, NE, USA), and expressed in mm^2^.

The content of assimilatory pigments and soluble protein from plants.

Assimilatory pigments were extracted in 80% acetone from 500 mg of leaves and the absorbance was read at 663.2 nm (chlorophyll a), 646.8 nm (chlorophyll b), and 470 nm (carotenoids) [21]. The content of each compound was calculated using equations described in Wellburn (1994) [22].

Soluble proteins were determined by the Bradford method, using a 3% extract and a calibration curve with bovine serum albumin, the results being expressed in mg of protein per gram of fresh material [23].

##### The Color Parameters of Wheat Grass

The MiniScan XE Plus produced by HunterLab, Reston, VA, USA was used. The studied parameters were L, a, and b. *L* represents lightness to darkness, 100 to 0, *a* represents redness to greenness—0 to 100 = red and −80 to 0 = green—and *b* is the yellowness and blueness—0 to 70 = yellow; −100 to 0 = blue [18].

The content of the bioactive compounds (flavonoids and polyphenols) and the antioxidant activity of wheat grass extracts.

The content of total flavonoid, polyphenols, and antioxidant activity was quantified according to Lobiuc et al. (2017) [24] using a 5% (*w/v*) ethanolic leaf extract that was left 24 h for extraction at room temperature. Briefly, for the analysis of total flavonoids, 0.25 mL extract was used for mixing and incubation for 5 min with 0.1 mL NaNO_2_, after which 0.15 mL AlCI_3_ was added and incubated for 6 min. At the end, 0.5 NaOH was added and the absorbance of the samples was read with a spectrophotometer at 510 nm. For the analysis of total polyphenols, 0.1 mL of extract was mixed and incubated for 5 min with Folin–Ciocalteu reagent, after which Na_2_CO_3_ 7.5% was added and incubated for 90 min and the absorbance of the samples was read at 760 nm. Total flavonoids were expressed in quercetin equivalent per g of fresh weight (µg QE g^−1^ f.w.) and total polyphenols were expressed as gallic acid equivalent per g of fresh weight (mg GAE g^−1^ f.w.) using a calibration curve. The antioxidant activity was expressed as % inhibition of DPPH (2.2-diphenyl-1-picrylhydrazyl, Sigma, Schnelldorf, Germany) [21].

##### Nutritional Composition of the Wheat Grass

Proximate composition of wheat grass (humidity, protein, ash, neutral detergent fiber-NDF, acid detergent fiber-ADF, fiber, and energy value) was determined with DA 7250 NIR Analyzer (Perten Instruments, Hagersten, Sweden). For each sample, three pooled samples were read in triplicate. Results were expressed as % [19].

#### 2.2.2. Wheat Seeds Characterization Techniques

##### Atomic Force Microscopy

The surface morphology of wheat seed coat samples was examined using the MultiMode NanoScope III a Controller (Digital Instruments Veeco Metrology Group, Santa Barbara, CA, USA) atomic force microscope in tapping mode. Topography and phase images were obtained at a scan speed of 0.5 Hz and a resolution of 512 pixels/line. The acquisition and offline analysis were performed using the NanoScope 531r1 software [25].

##### Scanning Electron Microscopy

The wheat seeds were analyzed by SEM-EDX using a Zeiss EVO MA15 microscope coupled with a Thermo Scientific NORANTM 7 EDX system. The analysis parameters were a 10 kV accelerating voltage with a probe current of 160 pA for the images and 1 nA for EDX analysis, at a working distance (WD) between 5 and 7 mm and a magnification of 500×. All the samples, both treated and untreated (control), were analyzed under the same conditions in variable pressure (VP) mode, at a pressure of 60 Pa [26]. SEM analysis was used to obtain images of the wheat seed coat morphology, and SEM-EDX elemental analysis was used to quantify the percentage content of C, O, Na, S, Cl, K, and Ca in the seed coat.

##### X-ray Computer Tomography of Wheat Seeds

The analysis was performed according to the method described in [27]. Briefly, a Nikon XT H 225 X-ray tomograph was used to analyze the wheat seeds. The X-ray tube in this equipment can reach a maximum power of 450 W and the spatial resolution of the tomographic images is around 3 μm, depending on the type of sample studied. To perform tomography on wheat seeds, the optimal parameters used for all the seeds were: 75 kV voltage, 50 mA the intensity of the electrical current, 2 s for the exposure, no filters were used, 360 projections/1 frame, and 11.82 μm voxel size. Images were reconstructed using the VGStudio MAX 3.0 software.

##### Particle-Induced X-ray Emission

Particle-induced X-ray emission (PIXE) technique was used to determine the elemental ratios of the wheat seeds. A 2.9 MeV proton beam was generated using the 3 MV Tandetron accelerator from IFIN-HH [28]. The characteristic X-rays energy emitted during the experiments were measured with a FAST SDD silicon drift detector (Amptek Inc., MA, USA). Each sample was positioned at normal incidence to the beam for 300 s and the current was kept under 1 nA. The samples were placed in a helium atmosphere, 20 mm from the vacuum window (Si_3_N_4_) and the beam alignment was performed using an automated sample positioning system that includes lasers and a video camera.

##### Statistical Analyses

In this study, IBM SPSS v20 (IBM Corp, Armonk, NY, USA) software was used for data processing and results were presented as means ± standard errors. The differences between variants were tested by ANOVA (*p* < 0.05) and Tukey test [29].

## 3. Results

This section is divided into two parts: one related to plasma source characterization—Section 3.1; and the second one dedicated to the characterization of seeds and grass—Section 3.2. It provides the reader with a concise and precise description of the experimental results, as well as their interpretation and correlation.

### 3.1. Plasma Source Electro-Optical Characterization

Plasma diagnosis methods, applied to low-temperature atmospheric pressure discharges, are usually associated with: the evaluation of the applied voltage; discharge current and total charge; the estimation of the electric power and energy of the discharge; the acquisition of the light emitted by the plasma and the identification; and the interpretation of the excited species from the discharge.

**Figure 2:**
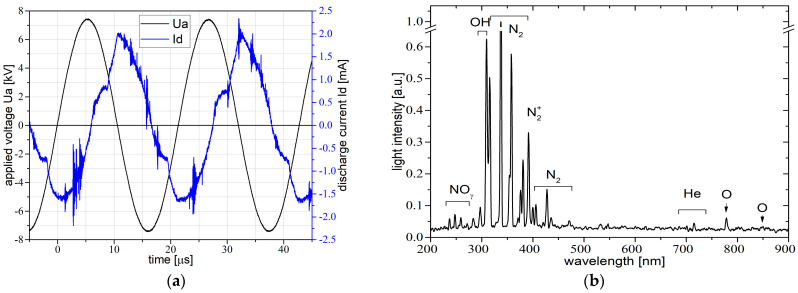
Plasma source electrical (**a**) and optical (**b**) characterization.

The electrical diagnosis of plasma was related to the applied voltage, discharge current, charge monitoring, and power/energy estimation. Typical voltage–current waveforms are depicted below in Figure 2a.

The electrical power over one period, estimated from the area of Lissajous figures, was around 0.5 W and a corresponding energy up to 11 µJ.

As depicted in Figure 2b, the used plasma source in this study has enough energy to excite, beside the working gas lines, helium lines, also some lines and bands of others atmospheric species, such as NO_γ_, OH, N_2_, N_2_^+^, and O. These are the so-called reactive species of nitrogen and oxygen (RONS), which can play a significant role in the plasma–sample-surface interaction.

Moreover, between 200 and 300 nm, the emission spectra of the discharge interacting with the wheat seeds was dominated by the NO_γ_ lines found at: 237, 247, 259, and 271 nm. Centered at 309 nm was the hydroxyl radical (OH) rotational band. Moving forward, from 315 to 390 were the bands of molecular nitrogen (N_2_): 315, 337, 357, and 375 nm. The nitrogen molecular ion band (N_2_^+^) was detected at 391 nm, which is important for estimating gas temperature using the Boltzmann plot method. These molecular nitrogen bands were encountered again between 400 and 470 nm. The working gas lines, helium lines in the 580–740 nm interval 588, 668, 706, and 727 nm were measured. According to Huzum et al. (2021) [11] and Nastuta et al. (2022) [10], the generation and excitation of N_2_ SPS (the second positive nitrogen system) and N_2_^+^ FNS (first negative nitrogen system) were based on the Penning effect of He metastables. Furthermore, lines of atomic oxygen at 777 and 845 nm were identified, corresponding to some metastable states (triplet states) of oxygen. The OH and O bands and lines that appear in the emission spectra are products of ambient O_2_ and H_2_O dissociation, as reported by [10,11,30,31,32,33] in similar plasma conditions. The presence of other excited species alongside helium lines (the working gas), suggests that these reactive oxygen and nitrogen species may and will take part in the wheat seed treatment. Secondly, the importance of such excited species is their usage in the estimation of gas temperature, as well as the vibrational temperature, both spectroscopic temperatures that can provide the investigator with information regarding the energetic species that can and will be used in the sample treatment.

### 3.2. Micro-Morphological Characterization of Wheat Seeds Exposed to Atmospheric Pressure Plasma

#### 3.2.1. AFM Surface Morphology

**Figure 3:**
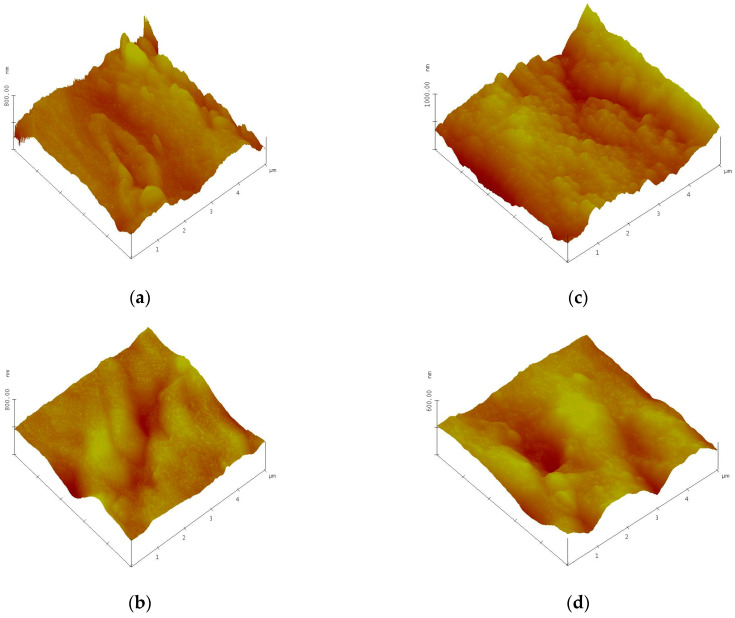
AFM 3D images of control wheat seeds (**a**,**b**) (‘Otilia’/’Dacic’) and plasma treated seeds (**c**,**d**) (‘Otilia’/’Dacic’); 5 × 5 µm^2^ scan size.

Figure 3 displays the 3D AFM topography of wheat seed samples.

The AFM images were made on a 5 × 5 µm^2^ area. For roughness analysis, the images were second order flattened and the R_q_ value was calculated using the software available with our microscope, NanoScope 531r1. It was found that root mean square (Rq) roughness calculated for 5 × 5 µm^2^ scan size decreases after the plasma treatment was performed. These results are summarized in Table 1.

#### 3.2.2. SEM-EDX Characterization

**Figure 4:**
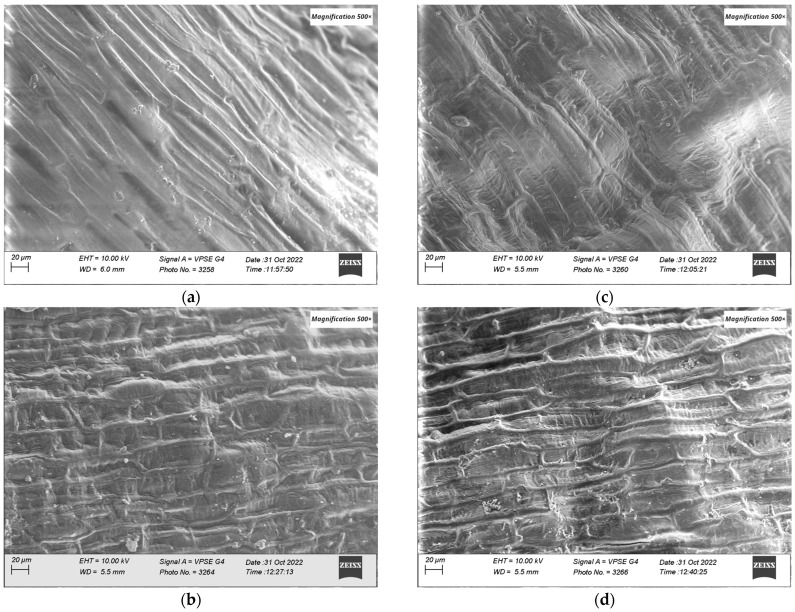
SEM images of control wheat seeds (**a**,**b**) (‘Otilia’/’Dacic’) and plasma-treated seeds (**c**,**d**) (‘Otilia’/’Dacic’); 500× magnification.

The morphology of the wheat seeds from the two cultivars was visualized before and after plasma treatments to see the possible effects on its surface. SEM images are presented in Figure 4. The wheat seeds were measured without any special preparation. In the images, it can be seen that, in the seeds treated with plasma, in both cultivars, the shape of the cells of the upper epidermis of the pericarp is much better defined than in the case of the control seeds, indicating that the plasma treatment produced an erosion effect at this level, thus making the surface much rougher.

SEM-EDX elemental analysis highlighted the presence and the atomic percent of C, O, Na, Cl, K, and Ca. For DT samples, Na, S, Cl, and Ca were below the detection limit, while for OC samples, only Ca was not detected. The oxygen concentration at the surface of the wheat seeds increased after plasma treatment with 4.44% for ‘Dacic’ and 2.74% for ‘Otilia’. These results are summarized in Table 2. This effect was also reported in the literature in [34].

#### 3.2.3. X-ray Computer Tomography

**Figure 5:**
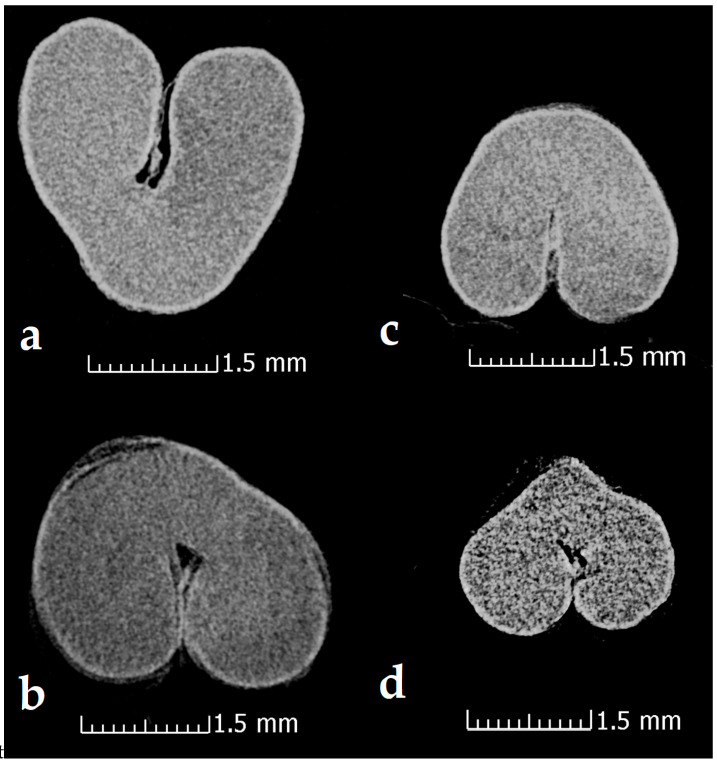
CT image images of control wheat seeds (**a**,**b**) (‘Dacic’/’Otilia’) and plasma-treated seeds (**c**,**d**) (‘Dacic’/’Otilia’).

**Figure 6:**
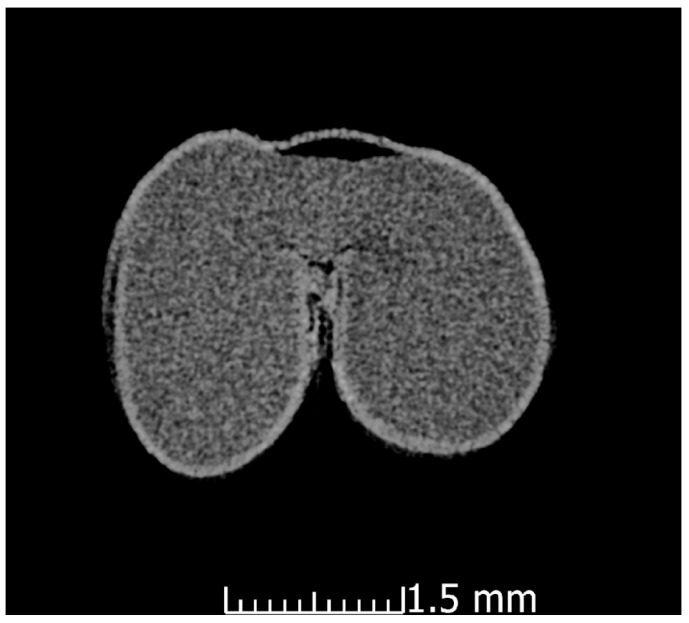
CT image of ‘Otilia’ cultivar wheat seed plasma-treated and swelling after PIXE measurement.

The X-ray computer tomography images of the wheat seeds from the two cultivars for wheat seeds before and after the plasma treatment were acquired in order to check for any possible effects in the internal structure of the wheat seeds (Figure 5). The thicknesses of the bran before and after the plasma treatment was also measured from the X-ray computer tomography images using the tool included in the analysis software (Table 3). The obtained values for the thicknesses agree with the literature data measured using SEM for ‘Otilia’ cv [35]. After analysis of all the images no conclusions can be drawn regarding the different density between the seeds that underwent the non-thermal plasma treatment and the control ones. Also, there is no considerable difference following the measurements made on the thickness of the outer layer of the seeds. What is clearly visible and is highlighted in Figure 6, is the swelling suffered by the wheat seeds that were subjected to PIXE analysis technique as a result of the interaction of protons with their surface, evaporating the respective area.

#### 3.2.4. PIXE Elemental Analysis

Elemental analysis was performed to check for any differences in the two cultivars. The collected spectra were analyzed using the GUPIX program configured in iterative matrix solution with an energy dependent H parameter. The following chemical elements were identified in both cultivars: Ca, Zn, Cl, Mn, Mg, P, S, K, Ca, and Fe. Table 4 presents the elemental ratios for Fe/Zn, Mn/Zn, and Ca/Zn. There is a clear difference between the two cultivars in terms of elemental concentration, with ‘Otilia’ having higher values than ‘Dacic’. Regarding the effect of plasma treatment, it induced an increase in the ratios for Fe/Zn, Mn/Zn, and Ca/Zn in both cultivars.

**Figure 7:**
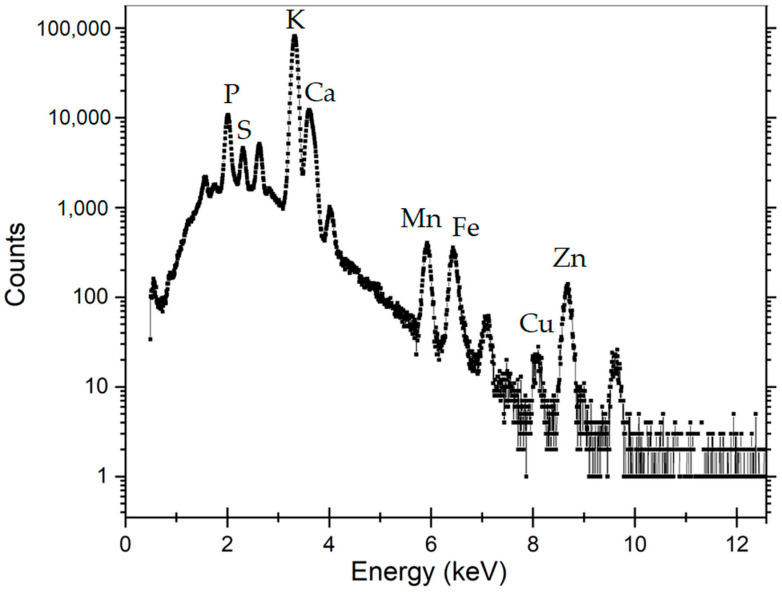
PIXE spectrum for ‘Otilia’ plasma-treated wheat seed.

Figure 7 shows the PIXE spectrum for Olivia cultivar wheat seeds and chemical elements identified.

### 3.3. Seed Germination and Plant Growth Parameters

The germination rate of wheat seeds was relatively high, over 90%, and was not significantly influenced by the plasma treatment (Table 5).

Regarding the growth parameters, the reaction of the plants to the plasma treatment was specific to each cultivar, with positively influenced values for ‘Dacic’ and negative for ‘Otilia’ (Table 5). The root length registered a significant increase of 25% for ‘Dacic’ treated compared to its control, and an insignificant decrease of 5% for ‘Otilia’ treated compared to its control. The plant height and fresh biomass were not significantly influenced by the plasma treatment, however, a small increase in fresh biomass by 9% for ‘Dacic’ treated and small decrease of 5% for ‘Otilia’ treated was observed. The biomass of ‘Dacic’ treated was significantly higher compared to both variants of ‘Otilia’. The total length increased significantly in ‘Dacic’ treated by 18% and decreased in ‘Otilia’ by 4%, but the difference was statistically insignificant. The leaf area increased by 13% in ‘Dacic’ treated (85.3 cm^2^) compared to the control (75.4 cm^2^) and decreased by 7% in ‘Otilia’ treated (79.3 cm^2^) compared to the control (73.5 cm^2^). Water content increased by 18% in ‘Dacic’ treated (79%) compared to control (77%) and decreased by 3% in ‘Otilia’ treated (74%) compared to control (78%).

### 3.4. Biochemical Composition of Wheat Grass

The content of assimilatory pigments and soluble protein from plants is shown in Table 6. The *chlorophyll a* content increased significantly in both treated cultivars, by 20% in ‘Dacic’ and 16% in ‘Otilia’. Additionally, the *chlorophyll b* content increased significantly in both treated cultivars, by 12% in ‘Dacic’ and 5% in ‘Otilia’, while the carotene content increased by 55% in ‘Dacic’- and 81% in ‘Otilia’-treated variants. The soluble protein content was influenced neither by the plasma treatment nor by the cultivar (*p* > 0.05).

Table 7 shows the values of the color parameters (L * lightness—darkness, a * redness—greenness, and b * yellowness—blueness). The statistical analysis of the parameters L, a, and b revealed that there were no significant (*p* > 0.05) differences between the treated variants and between cultivars.

The content of bioactive compounds (flavonoids and polyphenols) and the antioxidant activity of wheat grass extracts are presented in Table 8. The effect of plasma treatment was specific to each cultivar; thus, the content of flavonoids and polyphenols increased significantly for ‘Dacic’ treated by 63% and 32%, respectively, compared to the control variant, while the same parameters decreased for ‘Otilia’ treated by 13% and 12%, respectively, compared to the control. The antioxidant activity decreased in both treated cultivars, but the variations were insignificant (*p* > 0.05).

The nutritional composition of the wheat grass is shown in Table 9. The statistical analysis showed that there were no significant changes in the content of moisture, proteins, ADF, NDF and energy value. Only the ash content increased significantly in both treated ‘Dacic’ and ‘Otilia’ cultivars, by 27% and 25%, respectively, and the fiber content decreased significantly in ‘Dacic’ treated by 11% compared to the control.

## 4. Discussion

In this study, seeds from two varieties of wheat were exposed for 5 min to He cold plasma treatment. The seeds were subjected to a complex morphological and elemental characterization using AFM, SEM EDX, CT, and PIXE techniques. Through the AFM analysis, the surface of the wheat seeds could be studied and a moderate decrease in roughness was found after exposure to the plasma treatment. In the SEM images, the seed coat cells can be better observed after the application of the treatment, confirming the AFM results. Although the He-based plasma used in this study produced RONS (NOγ, OH, N_2_, N_2_^+^, and O), they did not have a strong destroying effect of the seeds surface seeds but rather contributed to a functionalization of the seed coat by a moderate and uniform erosion. The results in this study are different from those recorded by Starič et al. (2022) [36] who analyzed the surface of wheat seeds ‘Ingenio’ and highlighted through SEM a more nanostructured surface etching effect, while through AFM found an increase in roughness from 35 nm in the control to 80 nm after treatment with cold oxygen plasma for 90 s. The AFM and SEM techniques were also used by Saberi et al. (2022) [37] for the characterization of wheat seeds exposed to a 50 W plasma treatment for 150 s. The authors specified that they did not identify carvings on the seed surface of the control sample, but instead, based on the SEM images, the effects of ions and free radicals generated by the plasma treatments could be observed and causing an increase in seed hydrophilicity. Using SEM analysis, Waskow et al. (2021) [38] highlighted, on the one hand, an erosion effect of the *Arabidopsis thaliana* seed surface after exposure to cold plasma, while on the other hand, the AFM technique could not be used due to the seed’s deep contours and roundness. In this study, through the EDX module of the SEM, it was possible to identify the atomic percent of the elements C, O, Na, S, and Cl. There was a trend of increasing elemental concentration in ‘Dacic’ and decreasing in ‘Otilia’ after treatment, with a significant increase in O in treated ‘Dacic’ compared to its control. Elemental analysis was also performed through PIXE. The following chemical elements were identified in both cultivars: Ca, Zn, Cl, Mn, Mg, P, S, K, Ca, and Fe. It was found that there was a difference between cultivars and an increase in the Fe/Zn, Mn/Zn, and Ca/Zn elemental ratios after the plasma treatment of both cultivars. The results of this study are in agreement with those obtained by Cui et al. (2019) [39] who showed that the concentration of O increased in *Arabidopsis thaliana* seeds treated with plasma. An increase in the oxygen concentration after exposure of nasturtium seeds (*Tropaeolum majus*) to the plasma treatment was highlighted by Molina et al. (2018) [40] through XPS measurements. In the same study, the EDX analysis of elemental distribution maps showed an increase in K, while the distribution of other detected elements (S and P) remained the same or decreased [40]. Starič et al. (2022) [36] through SEM-EDX and XPS analysis reported a decreased amount of C and increased O concentration because of the formation of oxides, and an increased amount of K, S, Mg, and Ca due to selective etching. In this study, X-ray computer tomography analysis of wheat seeds revealed no significant modifications in seeds density and seed coat thickness between the plasma-treated and control; however, what could be highlighted was a swelling suffered by the wheat seeds that were subjected to PIXE analysis technique as a result of the interaction of protons with their surface, evaporating the respective area. The results of this study are in agreement with those reported by Srakaew et al. (2021) [41], who used a synchrotron radiation X-ray tomographic microscopy to study the broccoli seeds exposed to plasma treatments for 80 s and found no significant differences in the cross-sectional images between the treated and untreated seeds. However, the same author highlighted through SEM analysis that the seed coat was smoother after plasma treatment because the electrons and ion bombardment reduced the volcano-like protuberances, concluding that the plasma treatment does not affect the bulk characteristics of the seed but produces only fine modifications of their surface.

In this study, seed germination of the two wheat cultivars was not influenced by the plasma treatment. Moreover, a specific reaction of each cultivar in terms of initial growth was found; thus, growth parameters such as root length, plant height, fresh biomass, leaf area, and water content were positively influenced in the case of ‘Dacic’ and negatively in the case of ‘Otilia’. Short-term treatments with plasma can stimulate germination and initial growth through various mechanisms such as modifying the seed coat and increasing water absorption on the one hand, and on the other hand through the signaling action of RONS that activates the physiological mechanisms of exit from the dormancy stage [7,42,43]. Le et al. (2022) [8] suggested that the metabolic pathway through which plasma treatment stimulates root growth is through the upregulation of the gibberellic acid (GA3) hormone. However, the effect seems to be specific depending on the species and even the variety. For example, Starič et al. (2022) [36] showed that the germination rate decreased significantly in ‘Ingenio’ wheat cultivar treated with plasma for 90 s, while root length decreased and plant length and biomass were not influenced.

In this study, the biochemical analysis of wheat grass extracts showed a significant increase in the content of assimilatory pigments (chlorophyll a, chlorophyll b, and carotenoids). However, the analysis of the total soluble protein content and color parameters (L, a, and b) revealed that there were no significant differences between the treated variants and between cultivars. Stoleru et al. (2020) [44] showed that the content of assimilatory pigments also increased in lettuce treated with plasma-activated water as a result of the increase in NO_3_^−^ concentration, which is the main source of nitrogen for plants and which has an important role in the formation of chlorophyll. In this study, the seeds were directly exposed to cold plasma treatment before germination, so reactive N species do not have a direct role in plant nutrition. The increase in the chlorophyll content could be related to the changes produced by RONS in the interaction between air plasma and seeds [45]. On the one hand, the micromorphological changes at the seed level can determine a better absorption of water [37], and on the other hand, RONS can play a signaling role stimulating the physiological functions in seeds and plants. For example, Hasan et al. (2022) [45] measured a high H_2_ O_2_ content (3.56 µM g^−1^ FW) in the leaves of wheat plants sprouted from plasma-air-treated seeds and a marked increase in the activity of CAT and SOD enzymes, which are implicated in the antioxidant defense mechanism in plants, as well as an increased expression of TaCAT and TaSOD genes in roots of wheat. The authors suggested that the increased level of H_2_O_2_ can play a stimulating role triggering the physiological functions. As a result, the grain yield of wheat treated with plasma increased by 27.06%, and the quality of the obtained seeds improved by increasing the iron and fat content and decreasing the humidity [45].

Regarding the bioactive compounds, the effect of the plasma treatment was specific to each cultivar; thus the content of flavonoids and polyphenols increased significantly for ‘Dacic’ treated, while the same parameters decreased for ‘Otilia’ treated. The antioxidant activity decreased in both treated cultivars. According to Fernandes and Rodrigues (2021) [46] plasma treatments can increase the expression of PAL, C4H, and 4CL enzymes, which are involved in the biosynthesis pathway of the phenolic compounds, leading to an increase in these compounds.

In this study, the nutritional quality analyzed by FT-NIR of the wheat grass showed that for most of the quantified parameters (humidity, proteins, ADF, NDF, and energy value) no significant variations were recorded. Only the ash content increased significantly in both treated cultivars, and the fiber content decreased significantly in ‘Dacic’ treated. Direct treatments with plasma can stimulate the synthesis of some nutritional compounds, such as total amino acid content or isoflavones, as was demonstrated in the case of soybean sprouts [47].

As shown in this study, the positive effects of plasma treatments, whether on the initial growth parameters or biochemical and nutritional composition, are specific to each cultivar. Considering the variety of plasma parameters that can be tuned, such as applied voltage, repetition frequency, discharge current intensity, working gas, plasma species, and discharge geometry, this new field of plasma agriculture is both attractive to researchers and promising for farmers and society, as they are the beneficiaries of this new technology based on plasma [48].

## Figures and Tables

**Table 1:** R_q_ values for control and plasma-treated wheat seeds.

Samples	R_q_ (nm)
DC	116.8
DT	54.0
OC	90.0
OT	41.8

DC—‘Dacic’ control, DT—‘Dacic’-treated, OC—‘Otilia’ control, OT—‘Otilia’ treated.

**Table 2:** SEM-EDX elemental analysis.

Samples	C (at. %)	O (at. %)	Na (at. %)	S (at. %)	Cl (at. %)	K (at. %)	Ca (at. %)
DC	66.38 ± 0.39	32.78 ± 0.31	0.13 ± 0.02	0.12 ± 0.02	0.06 ± 0.01	0.06 ± 0.02	0.14 ± 0.02
DT	62.2 ± 0.35	37.22 ± 0.31	-	-	-	0.58 ± 0.02	-
OC	67.36 ± 0.42	31.42 ± 0.33	0.07 ± 0.03	0.09 ± 0.02	0.09 ± 0.02	0.97 ± 0.03	-
OT	64.73 ± 0.36	34.16 ± 0.29	0.10 ± 0.02	0.08 ± 0.02	0.05 ± 0.01	0.67 ± 0.04	0.21 ± 0.05

DC—‘Dacic’ control, DT—‘Dacic’-treated, OC—‘Otilia’ control, OT—‘Otilia’ treated. at. %—atomic percent. *N* = 3.

**Table 3:** Bran thickness for control and plasma treated-wheat seeds.

Samples	Thickness (μm)
DC	83.33 ± 3.33
DT	76.67 ± 8.82
OC	73.33 ± 14.53
OT	63.33 ± 3.33
Anova *p*-value	0.485

DC—‘Dacic’ control, DT—‘Dacic’-treated, OC—‘Otilia’ control, OT—‘Otilia’ treated. *N* = 3.

**Table 4:** PIXE elemental analysis.

Samples	Fe/Zn	Mn/Zn	Ca/Zn
DC	1.61	3.61	67.74
DT	2.45	3.65	78.34
OC	1.78	4.57	93.12
OT	2.78	4.64	109.85

DC—‘Dacic’ control, DT—‘Dacic’-treated, OC—‘Otilia’ control, OT—‘Otilia’ treated.

**Table 5:** Seed germination rate and plant growth parameters.

Samples	Germination (%)	Root Length (cm)	Plant Height (cm)	Total Length (cm)	Biomass (g)
DC	90.22 ± 0.28	11.63 ± 1.40 b	11.14 ± 1.16	22.77 ± 2.19 b	0.22 ± 0.01 ab
DT	90.67 ± 0.17	14.59 ± 1.22 a	12.47 ± 0.77	27.06 ± 1.84 a	0.24 ± 0.02 a
OC	90.67 ± 0.17	14.38 ± 1.01 a	12.09 ± 0.63	26.47 ± 1.49 a	0.20 ± 0.02 b
OT	90.22 ± 0.15	13.73 ± 0.98 a	11.68 ± 0.88	25.41 ± 1.55 ab	0.19 ± 0.01 b
Anova *p*-value	0.18	0.00	0.10	0.00	0.00

DC—‘Dacic’ control, DT—‘Dacic’-treated, OC—‘Otilia’ control, OT—‘Otilia’ treated. Different small letters represent significant differences according to Tukey test (*p* ˂ 0.05). *n* = 90 for germination and *n* = 45 for the rest of the parameters.

**Table 6:** The content of assimilatory pigments and soluble proteins in wheat grass.

Samples	Chlorophyll a (µg g^−1^)	Chlorophyll b (µg g^−1^)	Carotenoids (µg g^−1^)	Proteins (mg g^−1^)
DC	996.92 ± 6.38 c	375.44 ± 3.45 bc	146.29 ± 1.51 b	4.98 ± 0.00
DT	1196.93 ± 60 a	423.70 ± 3.13 a	228.11 ± 5.05 a	4.98 ± 0.00
OC	937.46 ± 7.46 d	366.30 ± 5.03 c	123.32 ± 0.90 c	4.98 ± 0.00
OT	1095.11 ± 0.68 b	386.77 ± 2.68 b	224.03 ± 0.50 a	4.98 ± 0.00
Anova *p*-value	0.00	0.00	0.00	0.19

DC—‘Dacic’ control, DT—‘Dacic’-treated, OC—‘Otilia’ control, OT—‘Otilia’ treated. Different small letters represent significant differences according to Tukey test (*p* ˂ 0.05). *N* = 3.

**Table 7:** The color parameters of wheat grass.

Sample	L *	a *	b *
DC	31.37 ± 0.64	−11.66 ± 0.23	22.56 ± 0.65
DT	31.23 ± 0.36	−11.48 ± 0.21	22.19 ± 0.27
OC	30.29 ± 0.4	−10.93 ± 0.13	22.97 ± 0.52
OT	31.13 ± 0.29	−11.52 ± 0.03	24.06 ± 0.56
Anova *p*-value	0.36	0.06	0.13

DC—‘Dacic’ control, DT—‘Dacic’-treated, OC—‘Otilia’ control, OT—‘Otilia’ treated. *N* = 3.

**Table 8:** The content of bioactive compounds (flavonoids and polyphenols) and the antioxidant activity of wheat grass extracts.

Sample	Flavonoids (mg QE g^−1^ fw)	Polyphenols (mg GAE g^−1^ fw)	DPPH (%)
DC	7.20 ± 0.77 b	3.59 ± 0.02 c	92.17 ± 2.04
DT	11.80 ± 0.40 a	4.78 ± 0.13 a	85.60 ± 1.46
OC	8.95 ± 0.70 b	4.14 ± 0.04 b	88.83 ± 1.41
OT	7.70 ± 0.08 b	3.61 ± 0.09 c	88.74 ± 0.78
Anova *p*-value	0.00	0.00	0.08

DC—‘Dacic’ control, DT—‘Dacic’-treated, OC—‘Otilia’ control, OT—‘Otilia’ treated. Different small letters represent significant differences according to Tukey test (*p* ˂ 0.05). *N* = 3.

**Table 9:** Nutritional composition of the wheat grass.

Sample	Humidity (%)	Protein (%)	Ash (%)	NDF (%)	ADF (%)	Fiber (%)	Energy (%)
DC	87.10 ± 1.09	20.70 ± 0.38	9.70 ± 0.12 bc	32.00 ± 0.58	44.00 ± 0.33	23.50 ± 0.09 a	10.99 ± 0.09
DT	84.10 ± 0.35	20.50 ± 0.26	12.40 ± 0.45 a	29.00 ± 1.45	44.00 ± 0.00	20.80 ± 0.35 c	10.82 ± 0.07
OC	84.80 ± 0.49	18.30 ± 1.13	8.70 ± 0.32 c	37.00 ± 2.33	45.00 ± 0.58	21.80 ± 0.12 b	10.67 ± 0.08
OT	84.00 ± 0.15	21.10 ± 0.66	10.90 ± 0.03 ab	27.00 ± 0.33	45.00 ± 0.00	22.50 ± 0.22 b	10.76 ± 0.05
Anova *p*-value	0.49	0.73	0.00	0.12	0.19	0.00	0.10

DC—‘Dacic’ control, DT—‘Dacic’-treated, OC—‘Otilia’ control, OT—‘Otilia’ treated. Different small letters represent significant differences according to Tukey test (*p* ˂ 0.05). *N* = 9.

## Data Availability

Data is contained within the article.
